# Relationship between Eating Alone and Handgrip Strength in Korean Older Adults

**DOI:** 10.3390/nu16050654

**Published:** 2024-02-26

**Authors:** Min Young Yoo, Hong Ji Song, Kyung Hee Park, Young-Gyun Seo, Hye-Ji An, Yu-Jin Paek, Hye-Mi Noh

**Affiliations:** Department of Family Medicine, Hallym University Sacred Heart Hospital, College of Medicine, Hallym University, Anyang 14068, Republic of Koreahongjisong5@gmail.com (H.J.S.); beloved920@gmail.com (K.H.P.); yg035@daum.net (Y.-G.S.); hjian9011@gmail.com (H.-J.A.); samsumok@gmail.com (Y.-J.P.)

**Keywords:** dietary intake, dynapenia, eating alone, handgrip strength, older adults, sarcopenia

## Abstract

Sarcopenia is defined as an age-related decline in muscle mass, muscle strength, and physical performance. Eating alone has been linked to various health issues in older adults. This study investigated the relationship between eating alone and handgrip strength (HGS) in older adults using data from 7278 individuals (≥65 years) who participated in the 2014–2019 Korea National Health and Nutrition Examination Survey. HGS was measured using a digital grip strength dynamometer, relative HGS was calculated by dividing HGS by body mass index, and dynapenia was defined as an HGS < 28 kg for men and <18 kg for women. Multivariable logistic regression analysis showed that women who ate two meals alone were more likely to exhibit dynapenia than those who never ate alone (odds ratio [OR], 1.3; 95% confidence interval [CI], 1.03–1.77). If the groups that never ate alone or ate one meal alone were combined as the reference group, the probability of dynapenia was higher in the combined groups that ate two or three meals alone (OR, 1.25; 95% CI, 1.04–1.50). No association was observed between eating alone and dynapenia in men. This suggests that eating alone is a modifiable related factor of dynapenia in older women.

## 1. Introduction

South Korea has a rapidly aging population. Currently, 9.5 million people, 18.4% of the total population, are aged 65 years or older [1]. Sarcopenia is the age-related decline in skeletal muscle mass, muscle strength, and the ability to perform physical activities, which can also lead to poorer health outcomes and increased susceptibility to various diseases. The global prevalence of sarcopenia in older adults is 10–16% [2]. In 2016, the *International Classification of Diseases* (Tenth Revision, Clinical Modification), listed sarcopenia as an independent condition and assigned it the code M62.84 [3]. The decrease in muscle strength, known as dynapenia, has a greater impact on mortality and physical disability than it does on the decrease in muscle mass [4]. This led the Asian Working Group for Sarcopenia to introduce the diagnosis of “possible sarcopenia,” defined by either low muscle strength or reduced physical ability only, in 2019, specifically to enable earlier intervention in primary or community-based healthcare [5,6]. Handgrip strength (HGS) has been suggested as a biomarker of aging. HGS is a good indicator of overall muscle strength, and it is correlated with the strength of other muscle groups [7]. Numerous studies have reported that a low HGS is associated with frailty, chronic diseases, cognitive impairment, and increased mortality in the geriatric population [8]. The measurement of HGS is relatively easy and non-invasive, and this test has been widely used for the screening of older adults who are at risk of physical disability [9]. Malnutrition and physical inactivity are risk factors for sarcopenia, and lifestyle interventions are the primary treatment measures [10,11]. Solitary eating habits, or eating alone, among older adults are associated with an increased risk of poor nutrition [12]. The previous studies on older individuals have reported that eating alone is associated with depression, cardiovascular disease, cognitive impairment, frailty, and increased mortality [13,14,15,16]. The number of older adults who eat alone increased during the coronavirus disease pandemic in 2019 (COVID-19) [17]. Eating alone can lead to a reduction in the motivation to prepare and eat meals. Commensality (the act of eating together) is considered as a social interaction through the sharing of food and the exchange of ideas and opinions during meal times, which can create friendships [18]. Older adults who eat alone tend to consume less-diverse food and are less socially active [19,20]; this social frailty has been associated with sarcopenia [21].

Based on these previous findings, we hypothesized that eating alone is a modifiable factor related to decreased muscle strength and aimed to evaluate the relationship between eating alone and HGS in older adults using a nationally representative sample of older adults in South Korea.

## 2. Materials and Methods

### 2.1. Study Population

Data were obtained from the Korea National Health and Nutrition Examination Survey (KNHANES) conducted by the Korea Disease Control and Prevention Agency (KDCA). The KDCA have carried out these surveys periodically since 1998 to assess the health and nutritional statuses of the Korean general population. The KNHANES includes a health examination, health interview, and nutrition survey. We used data from the 2014–2019 KNHANES, which collected HGS data. Among 47,309 participants, 9825 were aged 65 years or older, and therefore, met the inclusion criterion. Following the exclusion of 1055 participants with incomplete data on HGS, 723 participants with incomplete data on eating habits and 769 participants who reported having breakfast, lunch, and dinner less than twice a week, resulting in a final total of 7278 participants, were included in the analysis.

### 2.2. Eating Alone and Dietary Assessment

The participants were asked if they usually consumed breakfast, lunch, and dinner with someone else or ate alone in the last year. The participants were categorized based on each of the following responses for three meals: never ate alone, ate one meal alone, ate two meals alone, and ate three meals alone. A semi-quantitative food frequency questionnaire consisting of 112 items was developed and introduced to the nutrition survey due to its acceptable reproducibility and validity [22]. To assess dietary intake, the participants were asked to complete 24 h dietary recall with the help of a qualified nutritionist. Total energy and nutrient intakes were calculated using the National Standardized Food Composition Table developed by the Ministry of Agriculture and Rural Development. Data on total energy, protein, carbohydrate, fat, *n*-3 polyunsaturated fatty acid (PUFA), *n*-6 PUFA, calcium, iron, sodium, potassium, vitamin C, carotene, retinol, thiamine, riboflavin, niacin, and dietary fiber intakes were analyzed.

### 2.3. Handgrip Strength, Relative Handgrip Strength, and Dynapenia

HGS was measured during the survey by trained staff using the same digital grip strength dynamometer (TKK 5401; Takei Scientific Instruments Co., Ltd., Tokyo, Japan). The participants were instructed to stand upright and hold the dynamometer with their arms at their sides. They were then directed to grip the instrument as tightly as possible for 3 s. Three measurements were performed for each hand, with a minimum rest interval of 60 s between measurements; the highest HGS score was used in the analysis. Dynapenia was identified according to the guidelines issued in 2019 by the Asian Working Group on Sarcopenia, which defined dynapenia as an HGS of less than 28 kg for men and less than 18 kg for women [5]. Relative HGS was calculated by dividing HGS by body mass index (BMI).

### 2.4. Other Variables

Data were collected in a specially designed and equipped mobile examination center using a standardized environment and equipment. The participants were divided into two groups depending on their age: 65–74 years and 75 years or older. The monthly household income was divided into quartiles: lowest, middle, upper–middle, and highest. Self-reported medical history data were collected on 15 clinically diagnosed chronic diseases: cancer, ischemic heart disease, stroke, hypertension, dyslipidemia, diabetes, osteoarthritis, rheumatoid arthritis, osteoporosis, asthma, chronic obstructive pulmonary disease, thyroid disease, renal failure, cirrhosis, and depression. Multimorbidity was defined as the simultaneous presence of multiple conditions. Smoking status was classified as current (the participant has smoked more than 100 cigarettes in their lifetime and currently smokes), former (the participant has smoked more than 100 cigarettes in their lifetime and does not currently smoke), or never smoked (the participant has smoked less than 100 cigarettes in their lifetime and does not currently smoke). The participants who had consumed alcohol at least once per month in the last year were considered drinkers. Physical activity was assessed using the Global Physical Activity Questionnaire (GPAQ) that consists of three domains, which included occupation, leisure, and transport [23]. Regular aerobic exercise was defined as either 150 min of moderate-intensity exercise or 75 min of vigorous-intensity exercise per week [24]. Regular strength training was defined as engaging in strength training at least twice a week. Strength training included push-ups, sit-ups, pull-ups using bars, and lifting dumbbells or barbells [25]. Height and weight measurements were obtained from the participants wearing light clothing and no shoes. BMI was calculated by dividing weight by height squared (kg/m^2^). According to the guidelines of the World Health Organization of the Asia-Pacific Centre, we categorized BMI into three groups as follows: underweight, having BMI < 18.5 kg/m^2^; normal or overweight, having BMI 18.5–24.9 kg/m^2^; and obese, having BMI ≥ 25 kg/m^2^ [26].

### 2.5. Statistical Analysis

Data from the KNHANES were extracted using two-stage stratified cluster sampling. This involved dividing the target population into several strata and randomly selecting clusters from each stratum to form the sample. Data are expressed as estimated percentages (standard error) or estimated means (standard error). One-way analyses of variance were used to compare differences in continuous data between the groups. Chi-squared tests were used to compare differences in the categorical data between groups. Multivariable logistic regression analysis was used to assess the association between eating alone and dynapenia. In the final model, age, sex, BMI, household income, education, marital status, alcohol consumption, smoking status, aerobic exercise, resistance exercise, total energy intake, protein intake, and comorbidity were adjusted as confounding variables. All statistical tests were two-sided, and the significance level was set at *p* < 0.05. All statistical analyses were performed using SPSS software version 25.0 (IBM Corp., Armonk, NY, USA).

### 2.6. Ethical Approval and Informed Consent

This study used publicly available data from the KNHANES, which was originally approved by the Institutional Review Board of the KDCA (IRB: 2013-12EXP-03-5C, 2018-01-03-P-A, 2018-01-03-C-A). The KNHANES respondents provided written informed consent prior to participation in this survey.

## 3. Results

### 3.1. Characteristics According to the Frequency of Eating Alone

Table 1 shows the characteristics of the participants based on the frequency of eating alone. Of the 7278 participants, 3455 (47.5%) reported that they never ate alone, and 1588 (21.8%) reported that they ate three meals alone. Among the participants aged 75 years or older, those who ate three meals alone formed the largest group (49.2%), and those who ate one meal alone formed the smallest group (29.9%) (*p* < 0.001). The proportion of males tended to decrease as the frequency of eating alone increased, and it was highest in the group that never ate alone (56.5%) and lowest in the group that ate three meals alone (29.7%) (*p* < 0.001). The proportion of participants with a college-level education or higher was greatest in the group who never ate alone (13.5%) and lowest in the group who ate three meals alone (6.1%) (*p* < 0.001). The proportion of participants with the highest income level was greatest in the group who ate one meal alone (14.6%) and lowest in the group who ate three meals alone (5.9%) (*p* < 0.001). The proportion of current smokers was highest in the group who ate two meals alone (10.6%), and the proportion of alcohol drinkers was highest in the group who never ate alone (41.2%) (both *p* < 0.001). The participants in the group who ate three meals alone reported the lowest engagement in resistance (12.9%) and aerobic (27.4%) exercises (both *p* < 0.001) and the highest prevalence of multimorbidity (*p* < 0.001). The HGS and the relative HGS were greatest among those who never ate alone and gradually declined as the frequency of eating alone increased (*p* < 0.001). The prevalence of dynapenia was highest in the group who ate three meals alone (33.8%) and lowest in the group who ate one meal alone (22.6%) (*p* < 0.001).

### 3.2. Grip Strength According to the Frequency of Eating Alone by Sex

Due to the observed sex differences in the frequency of eating alone shown in Table 1, we compared the HGS indicators of the participants by sex (Table 2). For men, there was no significant difference in HGS, relative HGS, and the proportion of dynapenia among the different eating groups. However, for women, the HGS and relative HGS were the highest among those who never ate alone and the lowest among those who ate three meals alone (*p* < 0.001). Additionally, the prevalence of dynapenia was the highest in the group that ate three meals alone (33.8%) (*p* < 0.001).

### 3.3. Dietary Intake According to the Frequency of Eating Alone in Men

Table 3 shows the dietary intake of the participants according to the frequency of eating alone among men. The group that ate only one meal alone had the highest daily intake of total energy, carbohydrates, iron, potassium, niacin, and dietary fiber. Meanwhile, the group that ate two meals alone had the highest daily intake of protein, vitamin C, and riboflavin. The group that ate three meals alone had the lowest daily intake of total energy, protein, carbohydrate, iron, potassium, vitamin C, riboflavin, niacin, and dietary fiber.

### 3.4. Dietary Intake According to the Frequency of Eating Alone among Women

Table 4 shows the dietary intake of the participants according to the frequency of eating alone among women. The group that only ate alone one meal per day had the highest daily intake of total energy, fat, *n*-3 PUFA, *n*-6 PUFA, riboflavin, and dietary fiber. Conversely, the group that never ate alone had the highest daily intake of protein, carbohydrate, calcium, sodium, potassium, vitamin C, carotene, thiamine, and niacin. The group that ate three meals alone had the lowest daily intake of all the nutrients except iron.

### 3.5. The Proportion of Eating Alone among Participants with Dynapenia

**Figure 1:**
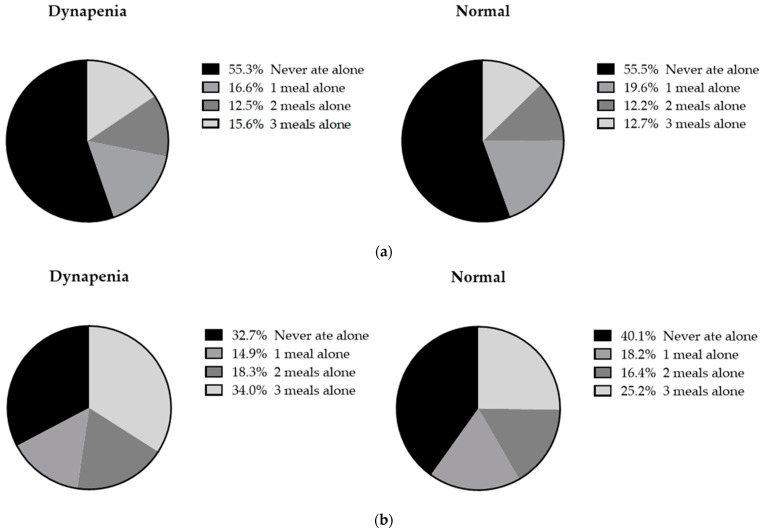
Comparison of the frequency of eating alone between participants with dynapenia and those without it, categorized as (**a**) men and (**b**) women.

Out of the 7278 participants, the prevalence of dynapenia was 26.8% (n = 1951). Among the participants with dynapenia, there were 695 (35.6%) men and 1256 (64.4%) women. Among the men, there was no significant difference in the proportion of eating alone based on the presence of dynapenia (*p* = 0.230) (Figure 1a). Among the women, those with dynapenia were more likely to eat two or three meals alone (18.3%, 34%) compared to those without dynapenia (16.4%, 25.2%) (*p* < 0.001) (Figure 1b).

### 3.6. Multivariable Analysis of the Association between Eating Alone and Dynapenia

Table 5 shows the multivariable analysis of eating alone and dynapenia. Among the men, there was no significant association between eating alone and dynapenia. Among the women, in Model 1, which was adjusted for age, the probability of dynapenia was higher in the individuals who ate two or three meals alone than in those who never ate alone (odds ratio [OR], 1.31 and 1.37, respectively; 95% confidence interval [CI], 1.03–1.67 and 1.11–1.70, respectively). In Model 2, which was adjusted for sex, BMI, household income, education, marital status, alcohol drinking, smoking status, aerobic exercise, resistance exercise, total energy intake, protein intake, and multimorbidity, the probability of dynapenia remained significantly higher in the individuals who ate two meals alone than it did in those who never ate alone (OR, 1.35, 95% CI, 1.03–1.77). However, in the group that ate three meals alone, the statistically significant association with dynapenia disappeared. If the groups who never ate alone or ate one meal alone were combined as the reference group, the probability of dynapenia was significantly higher in the combined groups who ate two or three meals alone than that in the reference group (OR, 1.25; 95% CI, 1.04–1.50).

## 4. Discussion

In this large representative sample of older individuals in South Korea, both the HGS and relative HGS were the highest among older women who never ate alone and the lowest among those who ate three meals alone. Eating two meals alone increased the likelihood of dynapenia compared with that caused by never eating alone. The older women in the combined groups who ate two or three meals alone had a higher probability of dynapenia compared to those who never ate alone or ate one meal alone.

Eating is an essential daily activity, and eating alone or with others is an important component of the Nutrition Screening Initiative (NSI) checklist for the screening of malnutrition in older adults [27]. However, studies investigating the direct relationship between eating alone and sarcopenia are lacking. Decreased HGS is a key component of sarcopenia and frailty in older adults. Therefore, our results are in line with those of the previous studies on older individuals, which reported that eating alone increases the risk of frailty. A Japanese study showed that eating alone, even if not living alone, is associated with frailty [28]. The longitudinal Korean Frailty and Aging Cohort Study, which monitored the participants for two years, found that a change from eating with others to eating alone increased the risk of weight loss and that sustained eating alone increased the risk of muscle weakness [16]. A recent Japanese longitudinal study conducted during the COVID-19 pandemic reported that eating alone increased the risk of weight loss compared to that of eating with others [17].

Eating alone is associated with a poor appetite [29], poor dental health, and oral frailty [30,31]. Older adults who eat alone may experience masticatory difficulties, leading to food avoidance and decreased dietary diversity [32,33]. Chemosensory variations refer to alterations in the senses of taste and smell, which commonly occur with aging. These changes might impact an individual’s perception of food flavors and aromas [34]. Older adults with reduced sensitivity to taste and smell might experience a decrease in their interest in food, which can lead to changes in eating habits. They might prefer simpler and less-pleasurable foods [35]. Meal preparation might be easier when eating alone. In this study, the association between eating alone and dynapenia was significant only in the older women, but not in the older men. There are some possible explanations for this sex difference. We found that older women eating three meals alone had the lowest daily intake of most nutrients. Especially, the dietary intake of *n*-3 PUFA, calcium, and carotene, according to the frequency of eating alone, was not different in the men, but was significantly lower in the women eating three meals alone. *N*-3 PUFA has anti-inflammatory properties and is used in preserving muscle protein [36]. A recent meta-analysis of 16 randomized controlled trials reported that *n*-3 PUFA supplementation improved both upper-extremity muscle strength and lower-extremity physical function [37]. Calcium is a regulatory signaling molecule for muscle fibers. Some cross-sectional studies showed that calcium intake is associated with lower odds of developing sarcopenia [38,39]. Carotene acts as an antioxidant in the body, and a higher intake of carotene is associated with better physical performance [40]. This suggests that eating alone is linked to a lower quantity and quality of food intake in older adults. Inadequate dietary intake may therefore be linked to the association between eating alone and decreased HGS. Although dietary protein intake was the lowest in both the men and women in our study, older women usually synthesize more muscle protein compared to that of older men [41,42]. It suggested that an increased rate of protein breakdown is related to more susceptibility to sarcopenia in women than men.

The previous studies have reported that eating alone is associated with adverse health outcomes in older adults. Eating alone increases the risk of depressive symptoms [43,44], and the Japan Gerontological Evaluation Study reported that a higher frequency of eating with others resulted in happiness in older adults [45]. In older women, eating alone has been associated with angina [15]. These conditions have also been associated with dynapenia [46,47]. However, in this study, eating alone was an independent factor associated with dynapenia, even after adjusting for multimorbidity, including depression. This suggests that eating alone is not only linked to dietary patterns, but is also associated with HGS. Our findings provide evidence of a relationship between eating alone and health outcomes among older adults.

However, this study has several limitations. First, because the 2014–2019 KNHANES did not measure the muscle mass or physical ability components of sarcopenia, we could not investigate the relationship between sarcopenia and eating alone. Second, despite the increase in the number of older adults who ate alone during the COVID-19 pandemic, there are no data on HGS after 2019 in the KNHANES. Therefore, we could not determine the impact of the changes in dietary habits caused by COVID-19 on the prevalence of dynapenia. Third, due to the cross-sectional study design, we could not prove a causal relationship between eating alone and the risk of dynapenia. Further studies are required to elucidate the association between eating alone and sarcopenia.

## 5. Conclusions

This study examined the relationships between eating alone, HGS, and dynapenia among older adults in South Korea. Older women who ate two or three meals per day alone were more likely to exhibit dynapenia compared to the individuals who never ate alone or ate one meal alone. This suggests that eating alone is a modifiable related factor of dynapenia in older Korean women.

## Figures and Tables

**Table 1:** Characteristics according to the frequency of eating alone.

Variable	Never Ate Alone	1 Meal Alone	2 Meals Alone	3 Meals Alone	*p*-Value
	(n = 3455)	(n = 1261)	(n = 974)	(n = 1588)	
Age					<0.001
65–74	59.9% (1.1%)	70.1% (1.5%)	64.7% (1.8%)	50.8% (1.5%)	
≥75	40.1% (1.1%)	29.9% (1.5%)	35.3% (1.8%)	49.2% (1.5%)	
Male	56.5% (0.7%)	49.5% (1.6%)	38.9% (1.8%)	29.7% (1.4%)	<0.001
BMI					0.211
<18.5	2.9% (0.3%)	2.9% (0.6%)	2.1% (0.4%)	3.3% (0.6%)	
18.5–24.9	63.0% (0.9%)	62.9% (1.6%)	59.7% (1.9%)	59.8% (1.5%)	
≥25	34.1% (0.9%)	34.2% (1.5%)	38.2% (1.9%)	36.9% (1.5%)	
Education attainment					<0.001
Less than elementary school	51.6% (1.2%)	50.2% (1.8%)	58.2% (2.0%)	69.2% (1.5%)	
Middle school	14.1% (0.7%)	17.4% (1.3%)	15.7% (1.4%)	13.1% (1.1%)	
High school	20.8% (0.9%)	19.8% (1.3%)	17.2% (1.6%)	11.6% (1.0%)	
College and higher	13.5% (0.9%)	12.5% (1.2%)	8.9% (1.1%)	6.1% (0.8%)	
Household income					<0.001
Low	42.0% (1.3%)	33.3% (1.7%)	40.9% (2.0%)	63.1% (1.5%)	
Lower middle	28.6% (1.1%)	31.3% (1.5%)	27.9% (1.7%)	20.9% (1.2%)	
Upper middle	17.0% (1.0%)	20.8% (1.5%)	18.4% (1.5%)	10.1% (1.0%)	
High	12.4% (0.9%)	14.6% (1.4%)	12.8% (1.4%)	5.9% (0.8%)	
Marital status (married)	99.9% (0.1%)	99.9% (0.1%)	99.0% (0.3%)	98.5% (0.3%)	<0.001
Smoking					<0.001
Never smoker	55.5% (0.9%)	59.7% (1.6%)	66.2% (1.9%)	70.5% (1.4%)	
Ex-smoker	36.0% (0.8%)	32.2% (1.5%)	23.2% (1.6%)	21.0% (1.2%)	
Current smoker	8.5% (0.5%)	8.1% (0.9%)	10.6% (1.2%)	8.5% (0.9%)	
Alcohol drinking	41.2% (1.0%)	39.2% (1.6%)	35.4% (1.9%)	28.8% (1.4%)	<0.001
Physical exercise					
Resistance exercise	22.3% (0.9%)	23.1% (1.5%)	20.9% (1.6%)	12.9% (1.0%)	<0.001
Aerobic exercise	35.5% (1.1%)	36.6% (1.7%)	37.0% (1.9%)	27.4% (1.3%)	<0.001
Multimorbidity					<0.001
≥3	19.5% (0.8%)	19.5% (1.3%)	23.3% (1.7%)	24.6% (1.3%)	
1–2	56.8% (1.0%)	57.2% (1.6%)	53.3% (1.9%)	57.7% (1.4%)	
0	23.7% (0.8%)	23.3% (1.4%)	23.4% (1.6%)	17.6% (1.1%)	
Handgrip strength	27.80 (0.18)	27.14 (0.30)	25.15 (0.34)	23.53 (0.26)	<0.001
Relative handgrip strength	1.18 (0.01)	1.15 (0.01)	1.06 (0.01)	0.99 (0.01)	<0.001
Dynapenia	23.5% (0.9%)	22.6% (1.4%)	28.7% (1.8%)	33.8% (1.5%)	<0.001

Data are presented as estimated percentages (standard error). *p*-values are derived from chi-square test. BMI, body mass index.

**Table 2:** Grip strength according to the frequency of eating alone by sex.

Variable	Never Ate Alone	1 Meal Alone	2 Meals Alone	3 Meals Alone	*p*-Value
	(n = 3455)	(n = 1261)	(n = 974)	(n = 1588)	
Men (n = 3273)	(n = 1902)	(n = 586)	(n = 357)	(n = 428)	
Handgrip strength	33.23 (0.20)	33.89 (0.32)	33.69 (0.45)	32.92 (0.41)	0.185
Relative handgrip strength	1.42 (0.01)	1.45 (0.01)	1.44 (0.02)	1.41 (0.02)	0.187
Dynapenia	20.9% (1.1%)	18.3% (1.8%)	21.4% (2.7%)	24.6% (2.5%)	0.230
Women (n = 4005)	(n = 1553)	(n = 675)	(n = 617)	(n = 1160)	
Handgrip strength	20.75 (0.15)	20.54 (0.21)	19.71 (0.22)	19.57 (0.18)	<0.001
Relative handgrip strength	0.87 (0.01)	0.86 (0.01)	0.81 (0.01)	0.81 (0.01)	<0.001
Dynapenia	26.8% (1.4%)	26.8% (2.0%)	33.3% (2.2%)	37.7% (1.8%)	<0.001

Data are presented as estimated means or percentages (standard error). *p*-values are derived from one-way analysis of variance or chi-square test.

**Table 3:** Dietary intake according to the frequency of eating alone among men.

Variable	Never Ate Alone	1 Meal Alone	2 Meals Alone	3 Meals Alone	*p*-Value
	(n = 1902)	(n = 586)	(n = 357)	(n = 428)	
Total energy (kcal/day)	1923.40 (20.27)	2019.57 (32.64)	1984.26 (48.18)	1866.34 (36.58)	0.009
Protein (g/day)	64.80 (0.87)	68.08 (1.26)	68.84 (2.05)	61.50 (1.72)	0.005
Carbohydrate (g/day)	322.26 (3.25)	334.96 (5.62)	330.07 (8.19)	312.69 (6.26)	0.046
Fat (g/day)	31.69 (0.72)	32.30 (0.96)	33.49 (1.50)	29.67 (1.28)	0.237
*n*-3 PUFA (g/day)	1.86 (0.07)	1.71 (0.07)	1.70 (0.11)	1.71 (0.12)	0.431
*n*-6 PUFA (g/day)	7.51 (0.19)	7.46 (0.24)	7.18 (0.33)	6.90 (0.30)	0.335
Calcium (mg/day)	513.81 (9.94)	499.64 (13.38)	516.81 (26.33)	472.96 (18.37)	0.226
Iron (mg/day)	14.60 (0.22)	15.27 (0.46)	14.61 (0.58)	13.20 (0.43)	0.008
Sodium (mg/day)	3528.61 (57.95)	3565.72 (91.63)	3527.35 (129.29)	3355.96 (112.79)	0.498
Potassium (mg/day)	3011.86 (41.53)	3040.96 (64.10)	2987 (94.83)	2726.54 (80.45)	0.008
Vitamin C (mg/day)	74.43 (2.11)	78.87 (4.66)	79.35 (6.79)	59.90 (4.26)	0.005
Carotene (µg/day)	3296.52 (116.97)	3451.12 (221.69)	3235.54 (224.90)	2959.15 (214.49)	0.409
Retinol (µg/day)	110.32 (17.06)	88.38 (8.22)	105.87 (12.89)	78.76 (12.10)	0.295
Thiamine (mg/day)	1.53 (0.02)	1.59 (0.04)	1.58 (0.05)	1.45 (0.04)	0.061
Riboflavin (mg/day)	1.33 (0.02)	1.35 (0.03)	1.37 (0.05)	1.20 (0.04)	0.021
Niacin (mg/day)	13.21 (0.20)	13.81 (0.31)	13.76 (0.53)	12.01 (0.37)	0.002
Dietary fiber (g/day)	28.14 (0.42)	29.24 (0.83)	27.27 (0.98)	25.23 (0.81)	0.003

Data are presented as estimated means (standard error). *p*-values are derived from one-way analysis of variance. Polyunsaturated fatty acid; PUFA.

**Table 4:** Dietary intake according to the frequency of eating alone among women.

Variable	Never Ate Alone	1 Meal Alone	2 Meals Alone	3 Meals Alone	*p*-Value
	(n = 1553)	(n = 675)	(n = 617)	(n = 1160)	
Total energy (kcal/day)	1529.06 (16.98)	1530.58 (33.12)	1430.10 (28.99)	1406.74 (21.35)	<0.001
Protein (g/day)	49.97 (0.74)	48.73 (1.11)	46.63 (1.02)	45.38 (0.88)	<0.001
Carbohydrate (g/day)	275.92 (3.09)	273.58 (6.19)	255.62 (5.67)	255.06 (3.79)	<0.001
Fat (g/day)	23.63 (0.53)	25.45 (1.08)	23.21 (0.81)	21.00 (0.67)	0.001
*n*-3 PUFA (g/day)	1.45 (0.05)	1.49 (0.11)	1.24 (0.08)	1.18 (0.71)	0.001
*n*-6 PUFA (g/day)	5.73 (0.14)	5.89 (0.24)	5.54 (0.23)	4.91 (0.18)	0.001
Calcium (mg/day)	411 (8.44)	408.44 (11.85)	391.20 (12.20)	377.67 (9.99)	0.049
Iron (mg/day)	12.07 (0.40)	12.07 (0.52)	10.40 (0.33)	10.68 (0.27)	0.001
Sodium (mg/day)	2681.33 (52.14)	2519.27 (80.32)	2380.37 (74.79)	2350.08 (58.40)	<0.001
Potassium (mg/day)	2557.63 (41.24)	2455.69 (64.18)	2327.35 (63.58)	2251.82 (46.76)	<0.001
Vitamin C (mg/day)	76.39 (3.29)	69.50 (3.63)	69.56 (4.53)	57.9 (2.28)	<0.001
Carotene (µg/day)	2915.32 (120.72)	2876.21 (149.12)	2472.45 (145.50)	2441.62 (126.11)	0.011
Retinol (µg/day)	61.27 (2.69)	85.27 (19.16)	74.18 (6.42)	61.11 (3.44)	0.162
Thiamine (mg/day)	1.23 (0.02)	1.19 (0.03)	1.12 (0.03)	1.12 (0.02)	0.001
Riboflavin (mg/day)	1.05 (0.20)	1.07 (0.31)	1.00 (0.31)	0.94 (0.23)	<0.001
Niacin (mg/day)	10.18 (0.17)	10.03 (0.25)	9.37 (0.25)	9.01 (0.19)	<0.001
Dietary fiber (g/day)	24.30 (0.43)	24.39 (0.78)	22.03 (0.67)	21.55 (0.50)	<0.001

Data are presented as estimated means (standard error). *p*-values are derived from one-way analysis of variance. Polyunsaturated fatty acid; PUFA.

**Table 5:** Multivariable analysis of eating alone and dynapenia.

Variable	Crude		Model 1		Model 2	
	OR	95% CI	OR	95% CI	OR	95% CI
Men						
Never ate alone		1		1		1
1 meal alone	0.85	(0.65–1.11)	1.10	(0.83–1.47)	1.03	(0.74–1.42)
2 meals alone	1.03	(0.74–1.43)	1.42	(1.01–1.99)	1.27	(0.84–1.93)
3 meals alone	1.23	(0.93–1.64)	1.26	(0.93–1.70)	1.12	(0.79–1.59)
Never ate alone or 1 meal alone		1		1		1
2 or 3 meals alone	1.18	(0.94–1.48)	1.29	(1.02–1.65)	1.18	(0.89–1.56)
Women						
Never ate alone		1		1		1
1 meal alone	1.00	(0.78–1.28)	1.08	(0.84–1.39)	1.03	(0.78–1.36)
2 meals alone	1.37	(1.08–1.73)	1.31	(1.03–1.67)	1.35	(1.03–1.77)
3 meals alone	1.66	(1.35–2.03)	1.37	(1.11–1.70)	1.20	(0.95–1.52)
Never ate alone or 1 meal alone		1		1		1
2 or 3 meals alone	1.54	(1.32–1.80)	1.32	(1.12–1.55)	1.25	(1.04–1.50)

## Data Availability

The data presented in this study are openly available from https://knhanes.kdca.go.kr/knhanes/main.do (accessed on 30 January 2024).
